# Incidental Findings in Neuroimaging: Ethical and Medicolegal Considerations

**DOI:** 10.1155/2013/439145

**Published:** 2012-11-20

**Authors:** Lawrence Leung

**Affiliations:** ^1^Centre of Neurosciences Study, Queen's University, 18 Stuart Street, Kingston, ON, Canada K7L 3N6; ^2^Centre of Studies in Primary Care, Queen's University, 220 Bagot Street, Kingston, ON, Canada K7L 5E9; ^3^Department of Family Medicine, Queen's University, 220 Bagot Street, Kingston, ON, Canada K7L 5E9

## Abstract

With the rapid advances in neurosciences in the last three decades, there has been an exponential increase in the use of neuroimaging both in basic sciences and clinical research involving human subjects. During routine neuroimaging, incidental findings that are not part of the protocol or scope of research agenda can occur and they often pose a challenge as to how they should be handled to abide by the medicolegal principles of research ethics. This paper reviews the issue from various ethical (do no harm, general duty to rescue, and mutual benefits and owing) and medicolegal perspectives (legal liability, fiduciary duties, Law of Tort, and Law of Contract) with a suggested protocol of approach.

## 1. Introduction

Modern scientific research often involves recruitment of human participants, and in the field of neuroimaging research employing various methods based on nuclear magnetic resonance, it is often a routine to obtain high-resolution structural scans of the brain and spinal cord as a template for subsequent interpolation of data. During such routine scans, it is not unusual to discover incidental abnormalities which are pure incidental findings not relevant to the actual research but can be life endangering to the participants if they are not pursued further. The question arises as to whether these otherwise healthy participants need to be informed, and if so, in what way, and finally, what needs to be done in the best interests of the participants to abide by the legal and ethical principles in research and medicine. Despite the prevalence of incidental findings of up to 10% in neuroimaging research, there is as yet a clear and unambiguous set of guidelines for dealing with incidental findings, and most researchers may not know how to deal with them [1]. A recent survey also showed that actual knowledge of the issue and logistics of management differ widely [2]. 

## 2. Case Scenario

FE, a 65-year-old lady who volunteered for an fMRI study for osteoarthritic knee pain, was incidentally found to have ischemic changes in the left temporal lobe during a base-line structural brain scan. She did not develop any neurological deficit, nor did she complain of any discomfort during the process of scanning. As the Principal Investigator of the study is a qualified clinician, he decided to withdraw FE from the study in her best interests. In view of her preexisting comorbidity of cardiac arrhythmias and diabetes, the PI decided to disclose the incidental findings to the participant in anticipation of further investigations and management. With the participant's permission, the PI established contact with the participant's family physician and related to him what happened, and the family physician agreed to offer close followup. FE developed neurological deficits 24 hours later and was subsequently admitted to the regional general hospital for repeat neuroimaging and treatment. A diagnosis of ischemic infarct of the left temporal lobe was confirmed and five days later, FE was discharged home after stabilisation with antiplatelet and anticoagulant therapy. She made a good recovery over the next six months with minimal loss of neurological functions. In retrospect, the PI and the research team felt distressed and unequipped in dealing with such incidental findings, in particular, clear documentation of the participant's wish whether to be informed of the incidental findings, a lack of standard guidelines and protocol, a lack of proper training in disclosing the findings in the best interest of the participants, and, finally, a lack of knowledge regarding the legal and ethical principles for disclosures. Also, it was agreed that the possibility of incidental findings should be dealt with more clearly in the consent form to safeguard the interests of either party. The team thence approached the Research Ethics Board for further advice and guidelines regarding these issues.

## 3. Incidental Findings during MRI Research: Incidence and Prevalence

A retrospective study of 151 magnetic resonance imaging (MRI) research scans revealed incidental findings up to 6.5% with higher preponderance in the elderly and females [3]. Another study reviewed retrospective brain MRI scans from 1000 volunteers and found incidental findings in 18%, of which 3% needing subsequent referral [4]. A cross-sectional study of structural MRI brain scans performed on 2000 participants from the general population in Netherlands revealed asymptomatic infarcts in 7.2% and primary tumours in 1.6% [5]. Another study from Germany looking at routine brain MRI scans from 2536 healthy young male applicants for military showed vascular and cystic abnormalities in 2.2% and intracranial tumours in 0.5% [6]. These two studies report tumours basing on their radiological appearances and hence reliable distinction between benign and malignant nature is not possible. A recent meta-analysis with 19559 patients from 16 studies with MRI of the brain found a lower figure, prevalence of 0.7% of neoplastic and 2.0% of nonneoplastic incidental findings [7]. In children, a retrospective study of 225 conventional scans in healthy pediatric participants involved in neurosciences research revealed incidental abnormalities in 21%, amongst which 1/3 required clinical referral [8]. That was in line with another study which reviewed 666 brain scans in pediatric participants from a neurology practice and found incidental findings in 25.7%, of which 8.7% were considered abnormal [9]. Overall, the prevalence of incidental findings in brain MRI performed in healthy volunteers and participants ranged from 2.0% to 18% for non-neoplastic abnormalities, and 0.5% to 3% for neoplastic lesions. Prevalence for similar incidental findings seems to be higher in children (21–27%, 1/3 needing further management), elderly, and females.

## 4. Consent Forms and Incidental Findings

A questionnaire survey sent to corresponding authors with peer-reviewed publication in 1991–2002 involving fMRI studies revealed great variability in the knowledge and existing protocols for handling incidental findings [2]. A recent study in Canada analysed a sample of 43 consent forms used for MRI and fMRI research and found 13 different strategies with varying attributes in dealing with incidental findings [10]. These attributes include participant(s)' choice to be informed of the finding, participant(s)' choice to inform their own physician for the findings, arrangement to inform the participants directly, arrangement to review the scans with a specialist, arrangement to follow up the participants, and so forth. There was also high variability of strategies used amongst different research centres and low consistency within a centre in using a single strategy. The authors opined that such variability cannot be solely ascribed to local Research Ethics Board (REB); rather, it is a combination of different research context and lack of consensus and common standards amongst all Canadian REBs. 

## 5. Policies around Disclosures

### 5.1. Disclosure of Risks

In Canada, the current legal standard of risks disclosure for research involving humans is full and comprehensive, in that all known and foreseeable risks—even rare and remote—must be disclosed to the research participants or their surrogate decision-maker. This is guided by two court decisions. In *Halushka* v. *University of Saskatchewan*, the Saskatchewan Court of Appeal ruled that the duty of disclosure of investigators to research participants is “at least as great as, if not greater than, the duty owed by the ordinary physician or surgeon to his patient” (1965), 53 D.L.R. (2d) 436 (Sask. C.A.). In *Weiss* v. *Solomon*, the Quebec Supreme Court established that all risks, even those rare and remote, must be disclosed to the research participant, especially if these risks could have serious consequences. The court in Weiss case based its decision on the disclosure requirements found in *Halushka* case as well as the declaration of Helsinki and the Civil Code of Quebec [11]. The declaration of Helsinki was released by the World Medical Association in 1964 [12] and is based on the Nuremberg code of 1947 [13] (the basis of tribunal indicting 23 Nazi physicians for their crimes for unethical human experimentation), which laid the three cornerstones for modern days ethical codes and regulations involving human research: voluntary informed consent, favourable risk and benefit analysis, and right to withdraw without repercussions (see the Appendix). In 2002, the Canadian Tri-Council Policy Statement on Research Involving Humans (TCPS) clearly stated that research participants must be provided with “a comprehensible description of reasonably foreseeable harms and benefits that may arise from research participation” (Art. 2.4c) [14]. Moreover, information regarding the “potential for physical or psychological harm” must also be provided to research participants (Art. 2.4 c) [14]. Even so, there was no mention of whether foreseeable incidental findings should be regarded as potential risks and harms, nor are there any clear guidelines as to when and what to disclose in the best interests of the participants. 

### 5.2. Disclosure of Incidental Findings

Even though incidental findings can be perceived as possible risks in any medical research, there are no clear policies or protocols specific for disclosure of incidental findings. During a National Institute of Health (NIH) funded workshop in 2006 [15], the following salient themes emerged as useful pointers for setting effective policies in disclosing incidental findings.

- A research protocol that provides for disclosure of suspicious incidental findings to participants is ethically and legally desirable.
- Researchers should respect the participants' autonomy and interests, and that includes prior informed consent for the right to know, or refusal of it, when incidental findings are detected.
- Researchers need proper training or, as an alternative, presence of qualified medical staff to detect and confirm the incidental findings before informing the participants.
- The possibility of false positives in incidental findings and their potential psychological harm to the participants needs evaluation and recommendations before being disclosed to the participants.
- Need to draw the line between legal and ethical responsibilities at the moment of consenting: whilst it would be legal and practical to inform the participants that the neuroimaging scans are for research purposes and not meant to detect abnormalities, and that the researchers are not trained to do interpret and give advice on them, it is arguably unethical to let a participant walk away with a possible brain tumour as suggested by the incidental findings unless such risks have been clearly communicated to the participants when they choose not to know about incidental findings.
- Need to standardise communication skills of the researchers and provide training for dealing with disclosure of incidental findings, which in essence, is an art of breaking bad news.
- Need for a good referral base for specialist opinion when incidental findings have to be pursued.
- Question of responsibility and management of incidental findings during subsequent secondary data analysis and sharing of database amongst the research team.
- Need for guidelines in informing third parties if the incidental findings suggest inheritable diseases that warrant genetic screening.

## 6. Ethical Principles

### 6.1. First, Not to Harm (*Primum Non Nocere*)

Scientific research insists on the same fundamental principle of *primum non nocere* (first, do no harm) as in medicine regarding any form of intervention given to the recipients. And when the intervention would carry any foreseeable degree of harm or risks, the researcher has the mandate to inform the participants, evaluate the harm and the risks of it, and monitor the participants over the course of research to avoid or mitigate the harm as much as possible. Most REBs would go to the last details to ensure that a clearly defined protocol exists to deal with risks and harm to safeguard the best interests of the participants. For incidental findings in neuroimaging research, this principle of *primum non nocere* will also motivate researchers to act appropriately in informing and managing the participants when the findings constitute foreseeable risks and harm. However, equally, the same principle will tend to suppress disclosure if the researchers think such findings are risk-free and harmless, as informing the participants invariably result in unnecessary worry or fear. In a similar way, this principle tends to discourage researchers in disclosing *all* known risks and possible incidental findings when obtaining consent for participation of study to avoid unnecessary alarm. Thus said, current consensus is to be an advocate of the participants and to inform and counsel them on incidental findings and their possibilities both at the consent stage and after actual identification. 

### 6.2. General Duty to Help and Rescue

**Figure 1:**
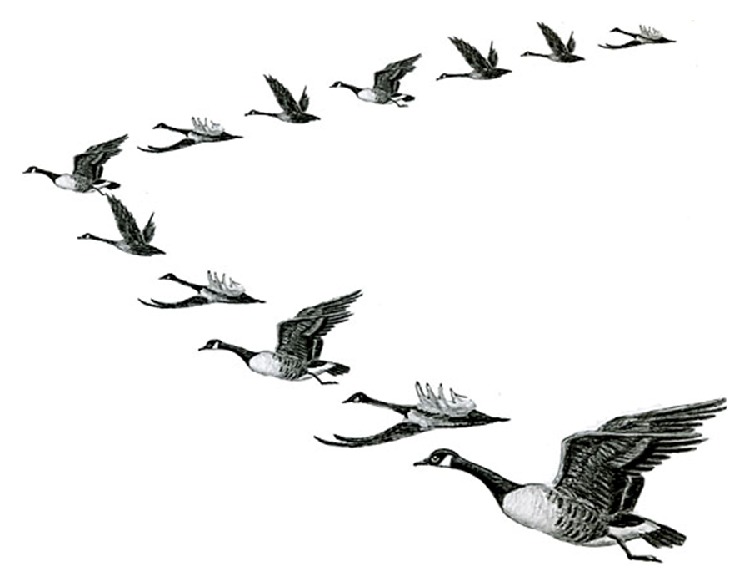
Special V-shape flying pattern of migrating Canadian geese exhibiting the duty of care. When one member is tired or sick, it will fall back and another member will stay behind to help and care.

There is perhaps little argument that the duty to help and rescue is hardwired in our human nature and psychosocial behaviour. We all see the duty to help a person in need whom no one else can help, albeit varying degree of sacrifice and risks of threats [16, 17]. Duty to help can be initiated as an individual or as a collective calling and will escalate to a duty of rescue in face of more urgent needs or disastrous situations, generally regarded as the Good Samaritan ethos [18]. Such duty to help or rescue does not require any prior acquaintance or professional relationships between the parties and has been suggested for consideration as part of the common law [19]. Scientist would even say it is a form of social behaviour when one member would help or nurse another infirm member without any hesitation, as well observed in community of ants, bees, and migrating geese (see Figure 1). One cannot underestimates the magnitude of driving force from human morals and conscience behind the duty of help and rescue, quoting from numerous examples that people went to extremes to save strangers in need and ended up losing their own lives. In context of neuroimaging research, incidental findings would invariably raise the question of potential life-threatening situations in the participants which would then compel the researchers' morals and conscience to act according to fulfil the duty to help or rescue. 

### 6.3. Mutual Benefits and Owing

When researchers and participants interact in conducting medical research, it is inevitable that a form of trusting relationships is formed, where the participants willingly submit themselves to the intervention protocol (or lack of it as controls) to accrue data for advancement of science and knowledge. To avoid any confounding or bias, research participants should theoretically enter the trial without an expectation of benefit as it is unclear if any benefit is possible with the given intervention. In reality, it is hardly the case. Participants often carry some expectations of benefits from the study, and researchers are often seen as the winner as they will gain new data and better understanding from what the participants go through in one way (as trial participants) or another (as control). Under such circumstances, the ethics of minimal mutual benefits may not apply in reality and the researchers may invariably feel indebted to the participants and “owe them something” in the process of research. In the context of neuroimaging, the researchers might be more inclined to look for incidental findings as a pay-back mentality to the participants, which in essence would not be compliant to the intention of the original research. It must be emphasized that researchers owe participants nothing except the duty of safeguarding them from harm and exploitation in the process of research [20], and the participants will reap their benefits in a communal and indirect way when their contributions lead to overall advance of science and medicine in our society.

## 7. Legal Principles

### 7.1. The Question of Legal Liability

In USA, the federal Common Rule provides a basic legal framework of accountability in medical research: minimizing overall risks, balancing research risks and benefits, description of the reasonably foreseeable risks and any benefits in the informed consent form, monitoring of research data, and the provision to the research participant of significant new findings that may affect the participant's willingness to continue in the study (45 C.F.R., 2007a–c). However, the Common Rule does not address the obligation to look for incidental findings, the processes that ensue discovery of suspected incidental findings, the appropriateness of seeking opinion for the findings and finally the scope of disclosure itself. Nor does it stipulate the best legal counsel to explain and consent participants on the possibility of incidental findings, the participants' right to refuse being informed, the legal and financial responsibility of subsequent followup for these findings, and the roles of funding agencies and the government in the process [21]. However, the issue becomes debateable when more data have now accumulated on the prevalence of incidental findings amongst neuroimaging research. Hence, the researchers are expected to have a better knowledge and awareness regarding foreseeable incidental findings, and whether that act of not detecting such incidental findings or not informing participants of such possibility would violate the issue of legal liability with reference to fiduciary duties, law of tort, law of contract, or law of bailment [22]. 

#### 7.1.1. Fiduciary Duties

In context of research, an implicit fiduciary relationship may arguably exist between the participant and the researcher, in that the participant (principal) have placed trust in the researcher (fiduciary) and the fiduciary has accepted the trust and agreed to act with undivided loyalty in the best interests of the principal [23], which may not be dependent on the signing of the consent form [24]. However, to constitute a case of breach of fiduciary duty for not seeking or not informing participants of incidental findings, one must need to assume full fiduciary relationship between researchers and participants (as in physicians and their patients) such that the researchers must forewarn participants of all foreseeable incidental findings to the best of their knowledge so as to fulfil the trust imposed by the participants [25]. Some legal scholars oppose the idea of imposing fiduciary duties to the researchers and they opine that clinical research is not interchangeable with clinical care, as the purpose of research is not to benefit the participants in a direct way [26]. At present, most court cases have ruled that full fiduciary duties are not to be imposed on researchers.

#### 7.1.2. Law of Tort

In the broadest sense, the Law of Tort deals with situations when behaviour or deeds of one person cause unfair losses or sufferings of another person without endangering our society. In the light of the Law of Tort, individuals are not obliged to warn of risks they did not create in the lack of special relationships or other exceptional circumstances. Due to the fact that incidental findings are not within normal expectations and are not part and parcel of the research process, the researchers should have no legal duty in forewarning participants. To establish negligence in the context of incidental findings, one must first establish a duty of tort on the researchers as a reasonable standard to seek and detect such incidental findings and fail to do so, or, in detecting the findings but fail to disclose them to the participants to guard their best interests. In USA, except for research performed within National Institute of Health (NIH), it is not mandatory to perform structural brain scan on neuroimaging participants. Hence, such tortious duty to detect and inform incidental findings as a reasonable standard cannot be established and imposed on the researchers, thereby the inability to prove negligence. However, the incidence of neuroimaging incidental findings of up to 3% for neoplastic lesions and 18% for non-neoplastic lesions [4] may provide a counterargument for this disposition. 

#### 7.1.3. Law of Contract

For breach of contract regarding incidental findings, there must be a prior contract established between researchers and participants delineating the action of detecting and disclosing such findings and the obligations to pay for damages should therefore be a failure to execute. The consent form signed by participants would usually be regarded as such a contract. However, it is common practice amongst most neuroimaging research institutes to structure the consent form in a risk-averse way to avoid possible litigation for breach of contract, using standard disclaimers like the following: the brain scans are for research purposes only; the brain scanning is not meant or designed for clinical diagnosis; the research team members are not trained in diagnosing brain pathology; the quality of research scans are not optimized for brain abnormalities. 

#### 7.1.4. Law of Bailment (Limited Entrustment)

Bailment describes a relationship in which physical possession of personal property (bailment) is transferred from one person (bailor) to another person (bailee) who subsequently took hold of the possession for a specific purpose within a limited time, with the consent to return possession of the property to the bailor when the purpose of the transfer has been accomplished. During the process, the bailee carries the duty of care of the bailment. A classic example would be someone sending a broken camera to the owner of a repair shop who agree to keep the camera until it is fixed, and during such consignment, should the repair shop be flooded, the owner of the repair shop has the duty to salvage the yet-to-be-repaired camera. In the context of incidental findings in neuroimaging research, the concept of bailment may be applicable as participants may have implicitly and partially entrusted researchers with certain aspects of their health, in knowing that there will be a review of the neuroimaging scans [27] and any abnormalities will be understood by the researchers with their superior knowledge, with the additional psychology that the researchers owe them some form of ancillary care [28]. To constitute a breach of law of bailment for incidental findings, one must demonstrate a lack of reasonable duty and care from the researcher with respect to the participant's health. The level of reasonable care and duty expected will be gauged by relative benefits from the bailment. In other words, if the participants have been informed and consent that the neuroimaging research is not designed and the researchers are not trained to detect abnormalities, failure to detect incidental findings or to inform participants of the findings will not be regarded as a reasonable care or duty, hence a weak support for breach of bailment.

## 8. Practical Approaches to Incidental Findings in Neuroimaging

**Figure 2:**
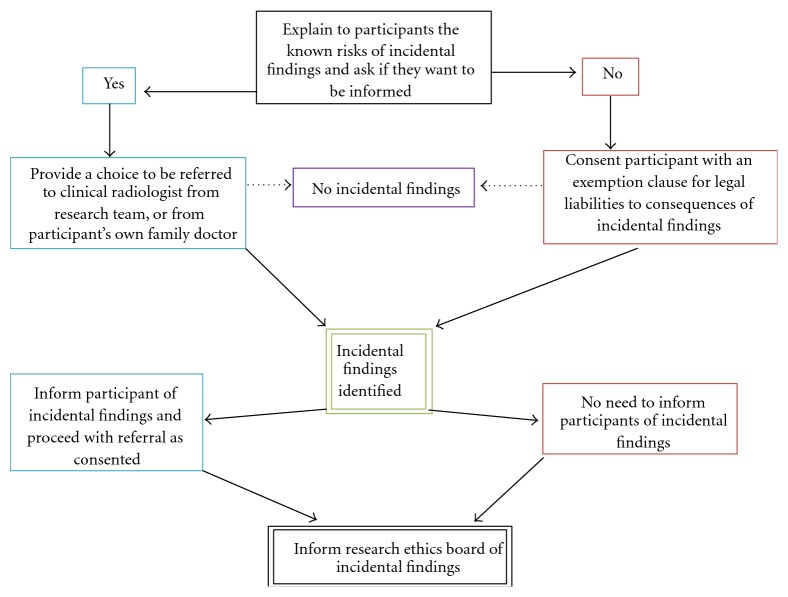
Protocol for managing incidental findings in neuroimaging.

Despite minor variations due to individual jurisdictions, the principles of dealing with incidental findings in neuroimaging are largely the same across major developed countries. When consenting participants for neuroimaging studies, the researchers have an obligatory role to address the possibilities of incidental findings as a known risk to the subjects and explain the protocol of management that will be taken, together with the need to inform the relevant research ethics board [21]. Thus said, there is no guarantee that every incidental finding will yield a true pathology that warrants clinical management, and pursuing every incidental finding will impose unnecessary costs to the researchers and possible detrimental anxiety to the subjects. A practical solution is summarised in Figure 2, where subjects will be asked during consent if they want to be informed of any incidental findings. If so, the full protocol of advising further expert opinions and facilitation of clinical care plus mandatory disclosure to the ethics board will be explained. The researchers will bear no legal responsibility to the actual pathologies and their consequences thereof if the subjects preferred not to be informed of the incidental findings. In reality, there might be a distinction in the type of incidental where participants will prefer to be informed or not, namely, whether it is life threatening or not. To respect confidentiality, a participant may decline the possible incidental findings of previous stroke or signs of brain atrophy that are not life endangering and hence the researchers are legally justified to respect the wish of not to be informed. However, the same subject may expect to be informed on a potentially fatal cerebral aneurysm which if not informed by the researcher will constitute an act of negligence. One can always refine the consent in allowing the subject to choose not to be informed if the incidental findings are unlikely to be harmful. Nevertheless, whether it is a detrimental finding often necessitates referral for specialist opinion and hence bringing the argument to full circle. In practice, the author's neuroimaging team has yet to consent a participant that has adamantly refused being notified of incidental findings. 

## 9. Conclusions

Magnetic resonance based neuroimaging research is one of the fastest growing fields in medicine especially with the advent of noninvasive tools like functional MRI. Though not compulsory, structural scans of the nervous system may be performed as part of the routine and in general incidental findings unrelated to the objectives of research have been detected in 2–18% of cases. Compared to the proposed intervention(s) which will be stringently assessed by Research Ethics Board for potential risks and harm according to standardised protocols, the issue of incidental findings lacks common consensus. In fact, recent data suggest an alarmingly high variability of strategies being used and low consistency of their execution. Some researchers may not know what to do whilst left to their own devices to cope. From the ethical standpoint, management of incidental findings relate to the principles of *primum non nocere*, duty of help and rescue, and the balance of mutual benefits versus owing. From the legal standpoint, one can approach incidental findings from the basis of common law, using the four perspectives of fiduciary duties, law of tort, law of contract, and law of bailment. In general, researchers are not legally obliged to diagnose or manage incidental findings in neuroimaging due to lack of proper training and resources; however, the participants should be clearly consented whether they would like to be informed of such incidental findings and what their options for subsequent management are if they preferred to be informed. Last but not least, globally endorsed guidelines and protocols for managing incidental findings, not only for neuroimaging but for general medical research, are still pending and urgently called for. 

## Appendix

### Excerpts from the Nuremberg Tribunal Forming the Basis of the Nuremberg Code [13]

“The great weight of the evidence before us to the effect that certain types of medical experiments on human beings, when kept within reasonably well-defined bounds, conform to the ethics of the medical profession generally. The protagonists of the practice of human experimentation justify their views on the basis that such experiments yield results for the good of society that are unprocurable by other methods or means of study. All agree, however, that certain basic principles must be observed in order to satisfy moral, ethical and legal concepts:

1. The voluntary consent of the human subject is absolutely essential. This means that the person involved should have legal capacity to give consent; should be so situated as to be able to exercise free power of choice, without the intervention of any element of force, fraud, deceit, duress, overreaching, or other ulterior form of constraint or coercion; and should have sufficient knowledge and comprehension of the elements of the subject matter involved as to enable him to make an understanding and enlightened decision. This latter element requires that before the acceptance of an affirmative decision by the experimental subject there should be made known to him the nature, duration, and purpose of the experiment; the method and means by which it is to be conducted; all inconveniences and hazards reasonably to be expected; and the effects upon his health or person which may possibly come from his participation in the experiment. The duty and responsibility for ascertaining the quality of the consent rests upon each individual who initiates, directs or engages the experiment. It is a personal duty and responsibility which may not be delegated to another with impunity.
2. The experiment should be such as to yield fruitful results for the good of society, unprocurable by other methods or means of study, and not random and unnecessary in nature.
3. The experiment should be so designed and based on the results of animal experimentation and knowledge of the natural history of the disease or other problem under study that the anticipated results will justify the performance of the experiment.
4. The experiment should be so conducted as to avoid all unnecessary physical and mental suffering and injury.
5. No experiment should be conducted where there is an a priori reason to believe that death or disabling injury will occur; except, perhaps, in those experiments where the experimental physicians also serve as subjects.
6. The degree of risk to be taken should never exceed that determined by the humanitarian importance of the problem to be solved by the experiment.
7. Proper preparations should be made and adequate facilities provided to protect the experimental subject against even remote possibilities of injury, disability, or death.
8. The experiment should be conducted only by scientifically qualified persons. The highest degree of skill and care should be required through all stages of the experiment of those who conduct or engage in the experiment.
9. During the course of the experiment the human subject should be at liberty to bring the experiment to an end if he has reached the physical or mental state where continuation of the experiment seems to him to be impossible.
10. During the course of the experiment the scientist in charge must be prepared to terminate the experiment at any stage, if he has probable cause to believe, in the exercise of the good faith, superior skill and careful judgment required of him that a continuation of the experiment is likely to result in injury, disability, or death to the experimental subject.”
